# Interprofessional collaboration in emergency department nursing care for patients with diabetic ketoacidosis: a narrative review

**DOI:** 10.3389/fendo.2026.1963622

**Published:** 2026-10-14

**Authors:** Lili Gao, Chengrong Li, Xue Liu

**Affiliations:** 1 Emergency Internal Medicine Department, Qinghai University Affiliated Hospital, Xining, Qinghai, China; 2 Emergency Intensive Care Unit (ICU), Qinghai University Affiliated Hospital, Xining, Qinghai, China

**Keywords:** care transition, diabetic ketoacidosis, emergency nursing, interprofessional collaboration, nurse-led pathway, patient safety

## Abstract

**Introduction:**

Diabetic ketoacidosis (DKA) is an acute metabolic emergency requiring timely risk recognition, fluid resuscitation, insulin therapy, electrolyte correction, dynamic monitoring, and coordinated care transitions. These interdependent tasks pose substantial challenges to emergency nursing. This narrative review aimed to synthesise current evidence on interprofessional collaboration in emergency DKA nursing and propose a conceptual framework to support coordinated care.

**Methods:**

A structured narrative review was conducted using literature retrieved from PubMed/MEDLINE, Embase, CINAHL, and the Web of Science Core Collection from database inception to July 10, 2026. DKA-specific clinical and implementation evidence was integrated with broader literature on interprofessional collaboration, communication, and healthcare implementation. The evidence was synthesised using Donabedian's structure–process–outcome framework.

**Results:**

Current collaborative approaches include nurse-led standardised pathways, risk-stratified subcutaneous insulin and non-intensive care unit management, collaborative medication management, laboratory and point-of-care monitoring, structured communication, digital decision support, and multidisciplinary discharge planning. Available evidence suggests that standardised collaborative pathways may improve the timeliness and consistency of care, shorten emergency department length of stay, and reduce intensive care resource use. However, evidence evaluating complete interprofessional nursing teams and nursing-sensitive outcomes remains limited. Based on the synthesis, a conceptual closed-loop collaboration framework comprising six interconnected stages was proposed: recognition and activation, shared risk stratification, coordinated monitoring and execution, escalation, care transition, and recurrence prevention.

**Discussion:**

Effective interprofessional DKA nursing requires shared clinical information, explicit responsibility, timely feedback, and predefined escalation procedures, with emergency nurses serving as continuous information integrators and process coordinators. The proposed framework provides an evidence-informed approach to organising collaborative emergency care. However, it has not been prospectively validated, and further implementation research is required to evaluate its feasibility, safety, and effects on nursing-sensitive and patient outcomes.

## 1 Introduction

Diabetic ketoacidosis (DKA) is a common acute metabolic emergency in people with diabetes and may involve ketoacidosis, intravascular volume depletion, electrolyte loss, and systemic risks related to precipitating conditions. International consensus statements and relevant guidelines on hyperglycaemic crises recommend that diagnosis and severity stratification incorporate blood beta-hydroxybutyrate, the degree of acidosis, circulatory status, level of consciousness, and comorbidities, and that the level of care—emergency observation, a general ward, or an intensive care unit (ICU)—be selected according to individual patient characteristics (1–4). The clinical presentation of euglycaemic DKA, particularly in patients receiving SGLT2 inhibitors or experiencing fasting, acute illness, or perioperative stress, further indicates that emergency nurses should not exclude DKA solely on the basis of blood glucose concentration. persistent vomiting, abdominal pain, Kussmaul breathing, dehydration, altered consciousness, insulin interruption, infection, and relevant medication use should all be incorporated into the initial assessment (5, 6).

Emergency nursing care for DKA is complex because multiple tasks must be performed concurrently while the patient’s condition changes rapidly. After triage, intravenous access, specimen collection, point-of-care monitoring, assessment of circulatory and neurological status, and identification of precipitating factors should be initiated promptly. Once treatment begins, blood glucose, serum potassium, blood ketones, acid-base status, urine output, cumulative fluid balance, and the patient’s clinical response require continuous reassessment. Any result may alter the sequence of subsequent tasks. For example, an abnormal potassium result may require medical reassessment and pharmacist review; a fall in glucose without resolution of ketosis may require renewed coordination of fluid and insulin plans; and before transfer, the team must confirm the next monitoring time, current infusions, and basal-insulin bridging. DKA care is therefore not a collection of independent procedures but a connected nursing process that requires continuous information exchange and confirmation of responsibility.

In the traditional model, physicians formulate the treatment plan and nurses execute orders sequentially, with interprofessional communication occurring mainly after an abnormality has been identified. Although this model remains necessary when the condition is complex and individualised decisions are required, it may lead to delays while awaiting orders, fragmented information, unclear responsibility during transfer, and omitted tasks in settings that require frequent reassessment and repeated adjustment. Interprofessional collaboration requires emergency nurses, emergency physicians, endocrinologists, clinical pharmacists, laboratory personnel, and diabetes specialist nurses to deliver continuous care around shared goals. Its value lies not in increasing the number of consultations but in ensuring that bedside changes, laboratory findings, prescribing risks, and self-management needs are translated promptly into accountable actions at the next stage of care.

A substantial proportion of DKA research has focused on comparisons of fluid types, routes of insulin administration, or individual biochemical markers. Such evidence is important for defining the clinical boundaries of treatment but provides less information on how nursing tasks are coordinated across professions, how responsibility is transferred, why pathway deviations occur, or how organisational conditions influence implementation. These questions are particularly relevant in the emergency department, where assessment, treatment, monitoring, escalation, and transfer often occur within a compressed period and depend on timely exchange of information between multiple professionals. This narrative review therefore focuses on interprofessional collaboration in emergency department nursing care for DKA across the patient journey, from early recognition and risk stratification to pathway implementation, multidisciplinary monitoring, escalation, care transition, discharge preparation, and recurrence prevention. The review first describes the literature-search and selection approach, then examines the theoretical basis and role configuration of interprofessional DKA care. Subsequent sections synthesise evidence on nurse-led and risk-stratified pathways, treatment safety and professional support, information exchange and digital collaboration, care transitions, and recurrence prevention, followed by a discussion of current limitations and priorities for future research. On the basis of this synthesis, we propose an evidence-informed closed-loop interprofessional collaboration framework to organise existing evidence and identify areas requiring prospective evaluation.

## 2 Theoretical framework and models of interprofessional emergency nursing for DKA

### 2.1 Literature search and selection

This narrative review was conducted based on a structured literature search. PubMed/MEDLINE, Embase, CINAHL, and the Web of Science Core Collection were searched from database inception to July 10, 2026. The search strategy combined terms related to diabetic ketoacidosis with terms related to emergency nursing and interprofessional collaboration. Major search terms included “diabetic ketoacidosis,” “DKA,” “emergency department,” “emergency nursing,” “interprofessional collaboration,” “multidisciplinary care,” “nurse-led,” “nurse-initiated,” “clinical pathway,” “quality improvement,” “clinical pharmacist,” “diabetes specialist nurse,” “laboratory monitoring,” “point-of-care testing,” “structured communication,” “care transition,” “discharge planning,” “readmission,” and “recurrence prevention.” Boolean operators and database-specific subject headings were used as appropriate. Eligible publications included clinical guidelines and expert consensus statements, systematic or scoping reviews, interventional and observational studies, quality-improvement and implementation studies, as well as qualitative and mixed-methods studies related to emergency DKA management, nursing practice,

interprofessional collaboration, treatment monitoring, care transitions, or recurrence prevention. Priority was given to studies directly involving patients with DKA and to recent evidence relevant to emergency or inpatient care. When DKA-specific evidence was limited, broader literature on interprofessional collaboration, nurse-initiated protocols, communication, and implementation science was appropriately included and regarded as indirect supporting evidence. Studies with limited relevance to emergency nursing processes or interprofessional collaboration were excluded. The reference lists of major guidelines, reviews, and eligible articles were also screened to identify additional relevant studies. The literature was synthesised narratively according to the major domains of the proposed framework, including risk recognition, pathway activation, multidisciplinary monitoring, risk escalation, care transition, and recurrence prevention.

### 2.2 Interdependence of emergency nursing tasks in DKA

Donabedian’s structure–process–outcome model provides a useful framework for explaining how multidisciplinary emergency nursing may influence DKA care (7). Structural factors include the nurse-to-patient ratio, emergency observation-unit capacity, point-of-care glucose and ketone devices, access to clinical pharmacists and diabetes specialist nurses, overnight laboratory support, and electronic information systems. Process factors include initial assessment, pathway activation, reassessment of key variables, management of critical values, task coordination, escalation of abnormalities, and interunit handover. Candidate outcomes for future evaluation may include metabolic correction, hypoglycaemia, hypokalaemia, and unplanned escalation to intensive care, as well as nursing workload, pathway acceptability, patient understanding of education, and readmission. Differences in the performance of the same pathway across institutions may reflect variation in structural resources and implementation processes rather than differences in treatment principles. Research on interprofessional collaboration suggests that team-based interventions can improve selected aspects of professional practice and care processes, although their effects are influenced by the organisational environment, team relationships, intervention format, and implementation fidelity (8). Studies of hospital communication have shown that professional hierarchy, role ambiguity, high-pressure working environments, and inconsistent communication methods can hinder information transfer, whereas shared goals, professional trust, and stable communication mechanisms facilitate collaboration (9). In DKA care, bedside observations, laboratory findings, medication review, treatment decisions, and nursing actions are closely interdependent. When each profession focuses only on its own tasks, gaps may occur, such as results being available but unacknowledged, treatment orders being changed without discontinuation of previous tasks, or patients being transferred without clear responsibility for the next scheduled assessment. Closed-loop communication provides an additional conceptual basis for the proposed framework. In healthcare teams, closed-loop communication generally involves transmission of information or a request, acknowledgement by the receiver, and confirmation that the message has been correctly understood or the requested action has been completed (10, 11). This approach is particularly relevant in emergency and other high-acuity settings, where several professionals must coordinate time-sensitive tasks. In the present review, the concept is extended from interpersonal communication to the broader care process. A clinical loop is considered complete when relevant information is recognised, communicated to the appropriate professional, translated into an accountable action, and followed by confirmation of task completion or reassessment of the patient’s response. This extension represents an author-derived application of established closed-loop communication principles to DKA care rather than an established DKA-specific model.

### 2.3 Integration of roles within the emergency interprofessional team

Roles within the emergency interprofessional team should be integrated according to the patient care pathway. During triage and early recognition, emergency nurses identify relevant symptoms and warning signs and obtain information on insulin interruption, pregnancy, fasting, infection, and relevant medication use. Emergency physicians establish the diagnosis and assess severity on the basis of clinical findings and laboratory results, while laboratory staff support timely processing of critical specimens. Patients with haemodynamic instability, altered consciousness, pregnancy, paediatric DKA, or complex cardiac or renal disease should receive early input from appropriate specialist or critical-care teams. During treatment and monitoring, nurses perform authorised interventions, conduct repeated bedside assessments, and verify completion of scheduled tasks. Physicians adjust treatment according to changes in clinical and biochemical findings. Pharmacists review medication-related and electrolyte-related risks, while laboratory staff provide timely results that may influence subsequent management. As the patient stabilises, collaboration shifts towards treatment transition, medication reconciliation, structured handover, assessment of self-management ability, and discharge preparation. Nurse-led care does not imply transfer of diagnostic or therapeutic responsibility to nursing staff. The actions that nurses may initiate, modify, withhold, or discontinue should remain within applicable regulations, professional scope-of-practice standards, institutional policies, individual competence, and locally authorised protocols (12–14). When predefined pathway-exit or escalation criteria are met, nurses may suspend routine protocol progression and request immediate medical reassessment. This does not imply independent discontinuation of clinically necessary treatment outside the nurse’s authorised scope. Clinical pathways should therefore clearly define which tasks nurses may initiate or adjust, which interventions require medical authorisation or independent double checking, which abnormalities require immediate escalation, and who assumes responsibility after escalation. Clear role boundaries and escalation procedures are essential to ensure that greater nursing involvement improves coordination without transferring inappropriate clinical risk to frontline nursing staff.

### 2.4 Closed-loop interprofessional collaboration model for DKA

Drawing on the structured narrative synthesis, we developed a conceptual closed-loop interprofessional collaboration framework for emergency DKA nursing care. The framework was derived through an author-led synthesis of DKA-specific clinical and implementation evidence together with broader literature on interprofessional collaboration and closed-loop communication. DKA-related care functions identified from the literature were grouped according to recognition, risk stratification, treatment, monitoring, escalation, care transition, and recurrence prevention. Overlapping functions were then consolidated into six interconnected stages, including recognition and activation, shared risk stratification, coordinated monitoring and execution, escalation, care transition, and recurrence prevention. The framework therefore represents an evidence-informed conceptual synthesis rather than a model derived solely from experiential reflection or a prospectively validated DKA care model. Within this framework, the emergency nurse acts as an information integrator and process coordinator but does not assume sole diagnostic or therapeutic responsibility. Nurses identify warning signs, contribute to risk stratification, integrate clinical and biochemical information, facilitate timely action, and coordinate handover and recurrence-prevention activities. When predefined pathway-exit or escalation criteria are met, routine protocol progression may be suspended and immediate medical reassessment requested. Such actions should remain within applicable regulations, professional scope-of-practice standards, institutional policies, individual competence, and locally authorised protocols. The six stages are interconnected rather than strictly linear. Information obtained during recognition and activation informs shared risk stratification, which in turn determines the required level of monitoring and care. New clinical or biochemical abnormalities identified during treatment may trigger reassessment and escalation. After a revised management plan has been established, the patient may re-enter coordinated monitoring at the same or a higher level of care. Care transition should occur only after treatment continuity, outstanding tasks, and monitoring responsibility have been confirmed. The framework therefore combines forward progression with feedback, reassessment, and escalation to form a dynamic closed-loop process, as illustrated in **Figure 1**. Several components of the framework are supported by DKA-specific studies, whereas the integrated six-stage structure and several nursing-sensitive measures represent an author-derived synthesis. Measures such as result-to-action time, critical-value closure rate, pathway deviation rate, escalation response time, and handover completeness should therefore be regarded as candidate process measures rather than established DKA-specific quality indicators. **Figure 1** illustrates the overall framework, while **Table 1** summarises the participants, nursing tasks, shared information, escalation triggers, and proposed process measures for each stage. For each time-based measure, **Table 1** specifies the starting event, ending event, principal data source, and threshold status. Where no validated DKA-specific time threshold is available, the interval should be analysed as a continuous process measure rather than assessed against an arbitrary benchmark. Where locally authorised protocols define specific monitoring or transition windows, these should be prespecified and reported as local protocol-based criteria rather than universal DKA-specific quality standards. Prospective studies are required before the framework can be used for formal implementation or quality assessment.

**Figure 1:**
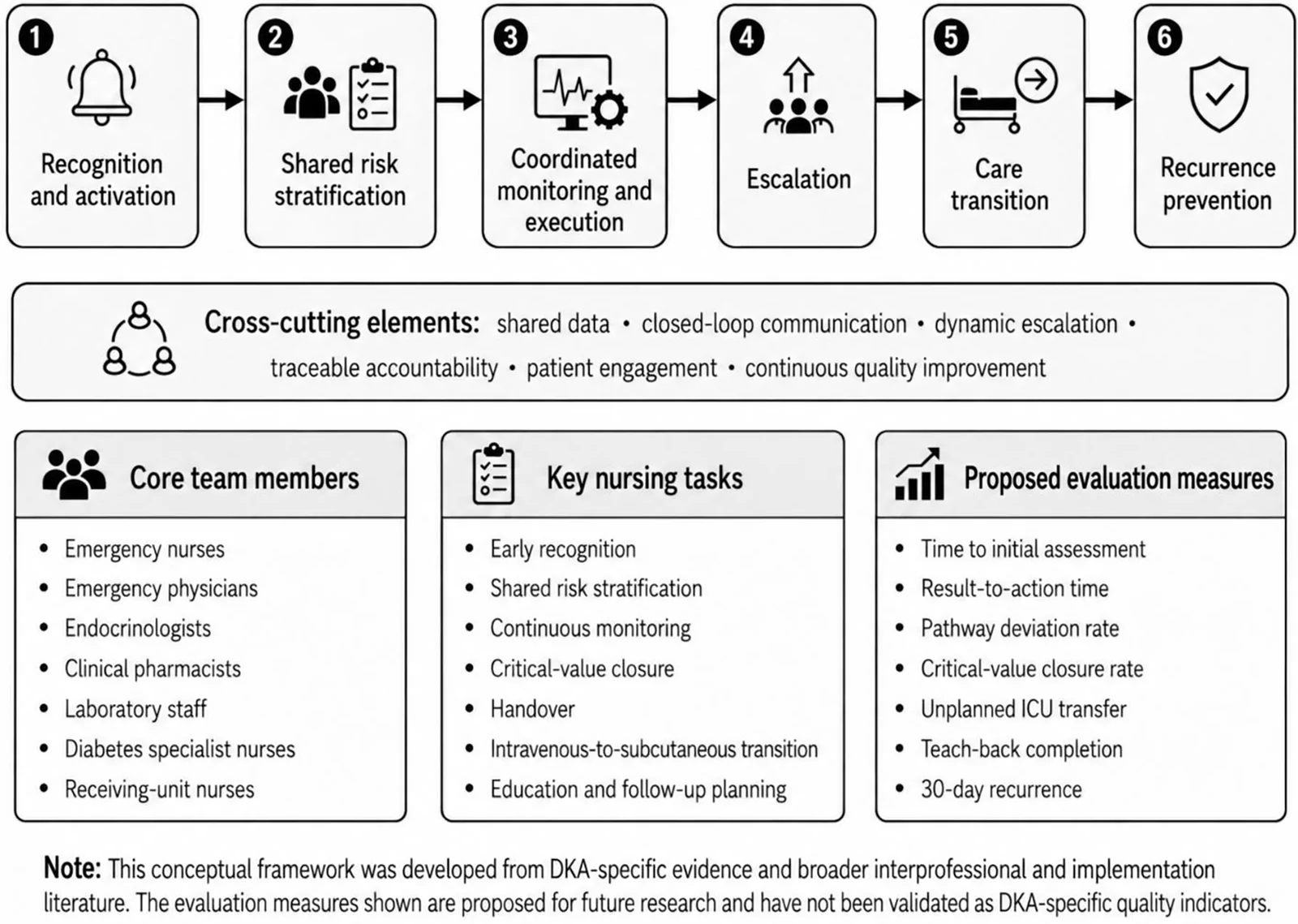
Closed-loop interprofessional collaboration model for emergency DKA nursing care. This conceptual framework was developed from DKA-specific evidence and broader interprofessional and implementation literature. The evaluation measures shown are proposed for future research and have not been validated as DKA-specific quality indicators.

**Table 1:** Stages and proposed process measures of the emergency DKA interprofessional closed-loop collaboration framework.

Stage	Core participants	Key nursing tasks	Shared information	Triggers for escalation	Proposed process measures and operational definitions
Recognition and activation	Emergency nurse, emergency physician, laboratory staff	Identify DKA warning signs, establish IV access, facilitate glucose, ketone, blood gas, and electrolyte testing	Symptoms, diabetes history, precipitating factors, glucose, β-hydroxybutyrate, pH, bicarbonate, potassium	Shock, altered consciousness, severe acidosis, severe hypokalaemia, pregnancy, paediatric presentation, oliguria, major cardiac or renal comorbidity	Time to initial assessment, from ED triage or registration to first documented DKA-relevant assessment. Starting event: ED triage timestamp; if unavailable, ED registration timestamp. Ending event: first documented DKA-relevant nursing or medical assessment. Data source: electronic triage record, ED registration record, and initial nursing/medical assessment record. Threshold status: no validated DKA-specific threshold; report as a continuous interval. Any institutional target should be prespecified and identified as a local criterion. Time to ketone or blood gas assessment, from documented suspicion of DKA or pathway activation to first assessment. Starting event: first documented suspicion of DKA or DKA pathway activation, whichever occurs first. Ending event: first documented availability of a blood ketone or blood gas result. Data source: EHR, pathway activation record, point-of-care testing record, and laboratory information system. Threshold status: no validated DKA-specific threshold; report as a continuous interval rather than against an arbitrary benchmark. Time to first fluid administration, from documented DKA recognition or pathway activation to initiation of the first prescribed intravenous crystalloid infusion. Starting event: documented DKA recognition or pathway activation. Ending event: documented initiation of the first prescribed intravenous crystalloid infusion. Data source: EHR/pathway record, medication administration record, and electronic infusion record. Threshold status: no validated DKA-specific threshold; report as a continuous interval. Any local treatment target should be prespecified and identified as a protocol-based criterion.
Shared risk stratification	Emergency physician, emergency nurse, endocrinology or critical-care team when required	Assess severity and match monitoring requirements with the appropriate level of care	Haemodynamic status, mental status, respiratory pattern, laboratory findings, monitoring capacity	Clinical deterioration, inadequate monitoring capacity, inability to provide timely escalation	Appropriateness of care setting, proportion of patients managed at a level of care consistent with predefined clinical and organisational criteria. Data source: clinical assessment record, admission/transfer record, and institutional level-of-care criteria. Threshold status: criteria should be predefined locally; no universal DKA-specific performance threshold is proposed. Unplanned ICU transfer rate, proportion requiring unexpected transfer to intensive care. Data source: EHR and transfer records. Threshold status: no universal DKA-specific performance threshold is proposed.
Coordinated monitoring and execution	Emergency nurse, physician, clinical pharmacist, laboratory team	Deliver prescribed treatment, monitor clinical and biochemical responses, identify abnormalities, confirm required actions	Fluid balance, urine output, glucose, potassium, β-hydroxybutyrate, acid-base status, current treatment	Persistent hypokalaemia, hypoglycaemia, fluid overload, poor ketone clearance, worsening acidosis	Result-to-action time, from availability of a predefined actionable abnormal result to documented clinical action. Starting event: verified release of a predefined actionable laboratory result or bedside availability of a point-of-care result. Ending event: first documented corresponding clinical response, including treatment initiation or adjustment, electrolyte replacement, repeat testing, medical reassessment, or a documented decision that no immediate intervention is required. Data source: laboratory information system, point-of-care testing record, EHR orders, medical/nursing notes, and medication administration record. Threshold status: no validated DKA-specific threshold; report as a continuous interval. Critical-value closure rate, proportion of critical results with documented acknowledgement, action, and reassessment. Data source: laboratory critical-value record, EHR, and nursing documentation. Threshold status: no validated universal DKA-specific performance threshold; definitions of critical values should follow institutional policy. Missed monitoring rate, proportion of scheduled assessments not completed within the predefined local protocol window. Starting event: scheduled due time for the required glucose, potassium, ketone, blood gas, or other assessment. Ending event: actual documented completion of the scheduled assessment. Data source: protocol/task schedule, nursing flowsheet, point-of-care testing record, and laboratory information system. Threshold status: determined by the locally authorised monitoring window; no universal DKA-specific time threshold is proposed.
Escalation	Emergency nurse, emergency physician, endocrinology or critical-care team	Suspend routine pathway progression when exit criteria are met, request immediate reassessment, intensify monitoring, prepare higher-level care when required	Clinical deterioration, mental-status change, worsening laboratory trends, treatment response	Haemodynamic instability, worsening consciousness, progressive acidosis, severe hypokalaemia, organ dysfunction	Escalation response time, from documented recognition of an escalation trigger to medical reassessment or initiation of escalation. Starting event: documented recognition of a predefined escalation trigger. Ending event: documented medical reassessment or initiation of escalation, whichever occurs first. Data source: nursing flowsheet, EHR alerts/clinical notes, medical orders, and rapid-response, transfer, or critical-care activation records. Threshold status: no validated DKA-specific threshold; report as a continuous interval. Any local response target should be prespecified. Unplanned ICU escalation rate, proportion requiring escalation to intensive care. Data source: EHR and transfer records. Threshold status: no universal DKA-specific performance threshold is proposed.
Care transition	Emergency and receiving-unit nurses, physicians, clinical pharmacist, diabetes specialist nurse	Complete structured handover, confirm treatment continuity, medication reconciliation, basal-insulin transition, and next monitoring task	Recent biochemical results, cumulative fluids, current treatment, insulin administration, next scheduled assessment	Delayed treatment transition, inadequate receiving-unit monitoring, clinical rebound	Handover completeness, proportion of transfers with all predefined mandatory DKA handover elements documented. Data source: structured handover record and EHR. Threshold status: mandatory elements should be predefined locally; no validated universal DKA-specific threshold is proposed. Basal-insulin bridging rate, proportion of eligible transitions completed within the locally specified protocol. Starting event: documented administration of basal insulin. Ending event: documented discontinuation of intravenous insulin infusion. Data source: medication administration record and electronic infusion record. Threshold status: the required interval should follow the locally adopted DKA/insulin-transition protocol and be prespecified; it should not be interpreted as a universal DKA-specific quality threshold. Time to first post-transfer assessment, from arrival at the receiving unit to first documented reassessment. Starting event: documented arrival at the receiving unit. Ending event: first documented clinical reassessment by the receiving unit. Data source: electronic transfer record and receiving-unit nursing or medical documentation. Threshold status: no validated DKA-specific threshold; report as a continuous interval.
Recurrence prevention	Diabetes specialist nurse, bedside nurse, physician, pharmacist, social worker, patient or family	Identify precipitating factors, assess self-management, confirm medication access, provide teach-back, arrange follow-up	Previous DKA, medication use, injection technique, health literacy, psychosocial risk, family support	Recurrent DKA, inability to access insulin, marked psychological distress, poor self-management capacity	Teach-back completion rate, proportion of eligible patients with documented completion of predefined education elements. Data source: nursing education record and discharge documentation. Threshold status: education elements should be predefined locally; no universal DKA-specific performance threshold is proposed. Follow-up completion rate, proportion completing planned follow-up. Starting event: hospital discharge. Ending event: documented completion of the planned follow-up encounter. Data source: discharge plan, outpatient EHR, referral record, or documented telephone/remote follow-up. Threshold status: the follow-up window should be prespecified according to the study or local care pathway; it is not an established universal DKA-specific quality threshold. 30-day DKA recurrence rate, proportion with documented recurrent DKA within 30 days after discharge. Starting event: hospital discharge. Ending event: documented diagnosis or readmission for recurrent DKA within 30 days. Data source: EHR, readmission record, and available follow-up records. Threshold status: the 30-day period represents a predefined outcome ascertainment window rather than a validated performance benchmark.

The measures listed in this table are proposed candidate process measures rather than validated DKA-specific quality indicators. For time-based measures, the starting and ending events should be predefined. Where no validated DKA-specific time threshold is available, the interval should be reported as a continuous measure rather than assessed against an arbitrary benchmark. For all time-based measures, the principal data source and threshold status are also explicitly defined. Where locally authorised protocols specify monitoring, treatment, transition, or follow-up windows, these should be prespecified and reported as local protocol-based criteria rather than universal DKA-specific quality standards.

## 3 Application of interprofessional collaboration in emergency DKA nursing pathways

### 3.1 Early recognition and risk stratification through interprofessional collaboration

Early recognition of DKA requires integration of symptoms, medical history, medication exposure, and point-of-care findings. Emergency nurses are often the first professionals to assess patients and should promptly identify potential precipitating factors, including insulin interruption, infection, reduced oral intake, persistent vomiting, pregnancy, and relevant medication use. When DKA is suspected, nurses should facilitate timely ketone, blood gas, and electrolyte testing and communicate abnormal findings promptly to physicians. Importantly, DKA should not be excluded solely because marked hyperglycaemia is absent. Baumer-Mouradian et al. (15) incorporated point-of-care testing, electronic order sets, and nursing and physician guidance into a paediatric emergency department quality-improvement programme. Following implementation, DKA determination time decreased from 86 to 26 minutes, point-of-care testing use increased from 0% to 98%, and unnecessary intravenous placement among patients without DKA decreased from 85% to 36%. These findings indicate that coordinated nursing assessment, timely bedside testing, and standardised pathways may improve early recognition and reduce unnecessary procedures. Similarly, Alsaedi et al. (16) reported that a collaborative programme involving simulation, feedback, and dissemination of best practices increased adherence to a nine-item paediatric DKA checklist from 77.8% to 88.9%, with significant improvements in hourly glucose monitoring and appropriate fluid administration. Although patient-level clinical outcomes did not improve significantly, the findings suggest that interprofessional interventions may improve care processes before measurable clinical benefits become evident. Risk stratification should consider both clinical severity and the monitoring and escalation capacity of the receiving unit. Selected patients with mild-to-moderate DKA may be considered for non-ICU management when frequent biochemical and clinical reassessment, adequate nursing capacity, and rapid medical escalation are reliably available. In contrast, haemodynamic instability, altered consciousness, severe acidosis, severe hypokalaemia, pregnancy, major cardiac or renal comorbidity, or inadequate monitoring capacity should prompt consideration of higher-acuity care. Safe placement therefore depends on matching patient risk with unit capability rather than on biochemical severity alone.

Euglycaemic DKA also requires particular attention because clinically significant ketosis and metabolic acidosis may occur despite only modest hyperglycaemia. This presentation is especially relevant in patients receiving sodium-glucose cotransporter 2 inhibitors and in those exposed to fasting, reduced carbohydrate intake, acute illness, dehydration, or perioperative stress. Medication reconciliation should therefore include current or recent SGLT2 inhibitor use, and suspected euDKA should prompt timely assessment of blood ketones and acid-base status regardless of glucose concentration. During care transition and discharge planning, the team should also confirm medication changes, oral intake, sick-day management, and the conditions for restarting SGLT2 inhibitors.

### 3.2 Nurse-led standardised pathways and team authorisation

The terms nurse-initiated and nurse-led describe related but distinct forms of nursing involvement and should not be used interchangeably. In this review, nurse-initiated care refers to predefined assessments or interventions that nurses are authorised to commence under an approved protocol without waiting for a new individual order, such as early screening, specimen collection, point-of-care testing, and scheduled monitoring. Nurse-led care describes a broader model in which nurses have an ongoing role in coordinating, implementing, monitoring, and evaluating an agreed care pathway within defined professional and organisational boundaries. Nurse-led pathways may therefore include nurse-initiated components but involve a more sustained role in pathway coordination and continuity of care. Evidence from protocol-based DKA care supports expanded nursing participation while also illustrating important distinctions among intervention models. Griffey et al. (17) implemented a computerised DKA protocol together with interprofessional education, audit, and feedback; selected process and safety indicators improved, although emergency department operational outcomes did not improve consistently. Lee et al. (18) evaluated a nurse-operated electronic insulin-infusion calculator in 181 adults with DKA and found reductions in hypoglycaemia and severe hypoglycaemia, without significant changes in DKA resolution time, insulin-infusion duration, or length of stay. Anis et al. (19) reported similar times to anion-gap closure with nurse-driven and physician-driven protocols, although the non-concurrent retrospective design and inter-centre differences in patient case mix and staffing limited conclusions regarding equivalence. Collectively, these studies indicate that nurse-operated, nurse-driven, nurse-initiated, and nurse-led approaches should not be treated as interchangeable models. Implementation also depends on organisational support. Gawthorne et al. (20) identified workload, psychological pressure, inadequate competence and confidence, documentation burden, and limited resources as barriers to nurse-initiated care, whereas education, clinical support, and positive nurse-physician relationships were facilitators. Kourouche et al. (21) similarly reported pathway complexity, insufficient clinical or managerial support, concerns about professional role boundaries, and increased workload across 29 emergency departments. These findings suggest that successful implementation requires clearly defined tasks and eligibility criteria, appropriate education and clinical supervision, leadership support, and ongoing audit and feedback rather than reliance on individual nurses alone. Both nurse-initiated and nurse-led pathways require explicit professional and organisational boundaries and should operate within applicable regulations, scope-of-practice standards, institutional policies, staff competence, and locally authorised protocols. When predefined pathway-exit or escalation criteria are met, routine protocol progression should be suspended and immediate medical reassessment requested. Candidate implementation-monitoring measures may include missed glucose or potassium assessments, delayed medication, incomplete critical-value communication, pathway deviations, and unplanned escalation. Available evidence is derived mainly from selected patients with mild-to-moderate DKA and should not be generalised to severe or complicated presentations. Nurse-initiated care therefore refers to protocol-authorised initiation of specified tasks, whereas nurse-led care refers to broader nursing coordination and oversight of an agreed pathway; neither substitutes for physician-led diagnosis or treatment decisions.

### 3.3 Risk-stratified Non-ICU pathways and interdepartmental quality improvement

The nursing significance of non-ICU DKA pathways lies less in demonstrating the superiority of a particular insulin regimen than in redistributing patients, nursing tasks, and ward resources. Bergmann et al. (22) used risk stratification, emergency–endocrinology collaboration, and standardised discharge criteria to reduce unnecessary hospitalisation among children with low-risk DKA. Yoo et al. (23) evaluated continuous insulin management for adults with mild-to-moderate DKA in medical-surgical wards or observation-level care and suggested that selected patients may be managed outside the ICU when monitoring requirements and timely physician response are clearly defined. These studies indicate that non-ICU care depends on strict patient selection, adequate nursing capacity, and reliable escalation mechanisms.

The Subcutaneous Insulin in Diabetic Ketoacidosis (SQuID) programme provides a representative example of interdepartmental implementation. The pathway was co-designed by emergency medicine, hospital medicine, endocrinology, pharmacy, emergency nursing, and ward nursing teams. Among 177 patients with mild-to-moderate DKA, emergency department length of stay was approximately 3 hours shorter than with conventional management, while adherence to prescribed point-of-care glucose monitoring remained high (24). SQuID II subsequently extended the pathway to additional eligible wards and reported high implementation fidelity together with reduced ICU utilisation (25). However, its single-centre design limits causal attribution and generalisability. A clinician acceptability study further showed that nurses were particularly concerned about timed injections, repeated blood sampling, fluid changes, and clustering of protocol-driven tasks, whereas physicians focused more on patient selection and treatment failure (26). These findings suggest that non-ICU pathways may redistribute rather than simply reduce nursing workload. Schneider et al. (27) further highlighted the importance of multidisciplinary co-design, targeted nurse education, electronic best-practice alerts, point-of-care materials, and ongoing consultation. From a nursing perspective, shifting eligible patients away from continuous intravenous insulin may reduce some infusion-related tasks and ICU resource use. However, scheduled insulin administration, repeated glucose and electrolyte monitoring, blood sampling, fluid adjustment, and reassessment remain time-sensitive nursing responsibilities. When staffing or workflow is inadequate, clustering of these tasks may increase the risk of delayed medication, missed monitoring, incomplete reassessment, or delayed recognition of clinical deterioration. Candidate measures for future evaluation of non-ICU pathways may include nurse-to-patient ratios, nursing time, missed or delayed care, task clustering, cognitive burden, and unplanned escalation, rather than relying solely on ICU utilisation or length of stay. Representative studies of interprofessional DKA nursing pathways and their nursing implications are summarised in **Table 2**. Current evidence applies primarily to carefully selected patients with mild-to-moderate DKA and should not be generalised to severe or clinically complicated presentations. Most available studies are observational, retrospective, or quality-improvement investigations. Reductions in ICU use or length of stay therefore should not be interpreted as evidence of equivalent safety or lower resource requirements across all DKA populations. Safe implementation requires both patient-level and organisational readiness. Candidates should generally be haemodynamically stable and should not have progressive alteration in consciousness, severe acidosis, severe hypokalaemia, major organ dysfunction, or other conditions requiring intensive monitoring or immediate specialist intervention. The receiving unit should be able to provide the required frequency of biochemical and clinical monitoring, timely medication administration, competent nursing staff, reliable point-of-care or laboratory support, prompt physician review, explicit escalation criteria, and rapid access to higher-acuity care. Non-ICU placement should therefore be based on a dual assessment of patient risk and unit capability. When these conditions cannot be reliably maintained, management in a higher-acuity setting may be safer.

**Table 2:** Representative Studies of Emergency Interprofessional DKA nursing and their nursing implications.

Study	Design/sample	Interprofessional or nursing model	Main findings	Nursing implication	Main limitation
Baumer-Mouradian et al. (15)	Paediatric ED quality-improvement study	Point-of-care testing + electronic order set + nursing/physician guidance	Selected key processes and ED operational indicators improved	Early recognition depends on coordinated nursing, medical, laboratory, and information-system support	Single-centre quality-improvement design
Alsaedi et al. (16)	Regional collaborative quality-improvement study	Simulation training + case feedback + best-practice checklist	Checklist adherence improved, whereas hard clinical outcomes changed little	Team behaviour often improves before patient-level outcomes become detectable	Limited power for clinical endpoints
Griffey et al. (17)	Before-after implementation study	Computerised DKA pathway + education + audit/feedback	Process variation and selected safety indicators improved	Pathway deployment requires ongoing education and feedback, not only order-set release	Non-randomised; multiple co-interventions
Lee et al. (18)	Retrospective study; 181 adults	Nurse-use electronic insulin calculator	Hypoglycaemia and severe hypoglycaemia decreased	Digital tools may reduce calculation errors and execution delays	Single-centre historical comparison
Anis et al. (19)	Multicentre retrospective comparison; 265 adults	Nurse-driven vs physician-driven protocol	Similar time to anion-gap closure between protocols	Supports the feasibility of nurse-authorised pathways under explicit boundaries	Non-concurrent comparison; centre heterogeneity
Gawthorne et al. (20)	Focus-group implementation study	Emergency nurse-initiated pathway implementation	Workload, confidence, documentation burden, and resources influenced uptake	Directly identifies frontline barriers and facilitators to pathway initiation	Qualitative design
Kourouche et al. (21)	Mixed-method study across 29 EDs	Implementation evaluation of nurse-initiated pathways	Pathway complexity, inadequate support, and increased workload were major barriers	Pathways should be simplified and aligned with frontline workflow	Not DKA-specific in all components
SQuID study group (22–27)	Cohort/prospective implementation/acceptability studies	Ward-based non-ICU pathway for mild-to-moderate DKA	ED length of stay decreased; implementation fidelity was acceptable; ICU use declined	Highlights the importance of multidisciplinary co-design, nurse training, and ward collaboration	Evidence mainly derived from selected patients with mild-to-moderate DKA; single-centre implementation and local staffing/resource conditions limit generalisability to severe or complicated DKA.
Olson et al. (28)	Retrospective study	Remote ICU pharmacist support	Laboratory monitoring was more frequent and DKA resolution occurred earlier	Pharmacists may function as medication-safety and transition-of-care nodes	Observational design cannot confirm causality
Periyasami et al. (29); Akiboye et al. (30)	Re-audit and systematic review	7-day specialist-nurse service/inpatient diabetes specialist nursing	Guideline adherence, knowledge, and satisfaction improved, but long-term evidence remained limited	Supports specialist-nurse involvement in continuity of care and discharge preparation	Long-term recurrence and cost data remain insufficient

## 4 Safety monitoring and professional support in interprofessional nursing

### 4.1 Continuous nursing assessment and closed-loop management of critical values

During DKA treatment, nursing safety depends not only on whether scheduled tests are completed but also on whether clinically important results are translated into timely action. Emergency nurses should integrate vital signs, level of consciousness, respiratory status, urine output, fluid balance, blood glucose, serum potassium, ketones, and current treatment status, and confirm that abnormal results have been reported and appropriately addressed. In this review, result-to-action time is defined as the interval from the availability of a predefined actionable laboratory or point-of-care result to the first documented corresponding clinical response. For central laboratory testing, the starting point is the verified release of the result. For point-of-care testing, it is the time at which the result becomes available at the bedside. The endpoint may include treatment initiation or adjustment, electrolyte replacement, repeat testing, medical reassessment, or a documented clinical decision that no immediate intervention is required. This measure differs from laboratory turnaround time, which primarily reflects the testing process from specimen collection or receipt to result reporting or verification. It also differs from door-to-treatment time, which mainly reflects the interval from patient arrival to initiation of a specific treatment. By contrast, result-to-action time reflects how efficiently available clinical information is translated into actual clinical action. For critical values, the complete closed-loop process should include result reporting, acknowledgement or read-back, clinical decision-making, nursing implementation, and post-intervention reassessment. A task should not be considered complete if any key step remains unconfirmed. It should be noted that result-to-action time is currently proposed only as a candidate process measure rather than a validated DKA-specific quality indicator and requires further prospective evaluation.

### 4.2 Support from clinical pharmacists, laboratory teams, and point-of-care monitoring

Clinical pharmacists may contribute to DKA care by reviewing high-alert medications, electrolyte replacement, renal-function-related risks, previous basal insulin use, medication-related precipitating factors, and treatment transitions. In a retrospective study, Olson et al. (28) found that patients receiving patient-specific interventions from a tele-ICU pharmacist underwent more frequent laboratory monitoring and achieved DKA resolution approximately 7.32 hours earlier than patients who did not receive the intervention. However, this intervention was delivered remotely and should not be considered equivalent to continuous face-to-face pharmacist participation. Remote pharmacist support may extend specialist availability and facilitate centralised medication review across locations, whereas face-to-face collaboration may allow more immediate bedside communication, direct participation in team discussions, and closer integration with local workflow. Olson et al. did not directly compare these two modes of collaboration, and their relative effects therefore remain uncertain. Because pharmacist involvement may have affected prescribing, monitoring, and team response at the same time, the findings show an association rather than a causal effect. Laboratory teams are also part of the treatment process because potassium, ketone, and acid-base results can alter monitoring intensity and subsequent management. Emergency and laboratory departments may agree on specimen priority, expected turnaround time, critical-value reporting, and the person responsible for receiving the result. Point-of-care testing can shorten the interval to result availability, but this benefit depends on device quality control, staff training, confirmation of unexpected results, and clear responsibility for acting on the information. A rapid test has limited value if the result is not reviewed or incorporated into treatment decisions.

### 4.3 Diabetes specialist nursing and nursing-sensitive outcomes

Diabetes specialist nurses can contribute across acute treatment, care transition, and preparation for self-management. Periyasami et al. (29) re-audited 126 DKA admissions after enhanced guideline education and introduction of a seven-day inpatient diabetes specialist-nurse service. Eleven of 18 audit standards improved, and 98.4% of patients received at least one specialist-nurse assessment during admission. Iatrogenic hypoglycaemia and transient hypokalaemia still occurred in 10.3% and 31.7% of admissions, respectively, showing that specialist-nurse involvement does not remove treatment-related risk. Akiboye et al. (30) reviewed 10 studies of inpatient diabetes specialist nurses. Most studies reported shorter hospital stays, and some reported fewer medication errors and improvements in patient knowledge or satisfaction. Evidence for mortality, readmission, and longer-term outcomes remained limited. In DKA care, specialist-nurse input may include assessment of precipitating factors, previous insulin use, injection technique, health literacy, medication access, and psychosocial risk, rather than being confined to education immediately before discharge. Standardised nursing terminology systems may strengthen the disciplinary basis for defining nursing interventions and nursing-sensitive outcomes. The Nursing Interventions Classification (NIC) provides standardised terminology for nursing interventions, whereas the Nursing Outcomes Classification (NOC) provides a structured framework for evaluating nursing-related outcomes (31, 32). In DKA care, these systems may support more consistent description of interventions such as surveillance, medication management, patient education, and care coordination, together with corresponding patient and care outcomes. Nursing-sensitive evaluation in DKA may include structural, process, staff, and patient-related domains. Candidate domains include timeliness of care, execution of scheduled tasks, nursing workload, team functioning, patient self-management, and recurrence (**Table 3**). These proposed domains are conceptually informed by standardised nursing terminology but have not been formally mapped to or validated against NIC or NOC. These domains are proposed for research and implementation evaluation and should not be interpreted as an established DKA-specific quality-indicator set. Staff competence is also relevant to pathway safety. Kajembula et al. (33) identified knowledge, procedural skills, and pathway accessibility as facilitators, whereas inadequate specialist training, limited nursing autonomy, nurse-physician disagreement, delayed laboratory results, and shortages of staff and equipment were barriers. Alskafi et al. (34) reported that 61.2% of 178 critical care nurses had a low level of DKA-related knowledge. Although the findings may not be directly transferable across settings, they support competence assessment, case-based training, simulation, and periodic refresher education.

**Table 3:** Proposed core outcome domains for emergency interprofessional DKA nursing research.

Domain	Candidate measures
Structural indicators	Nurse-to-patient ratio; emergency observation-unit capability; point-of-care glucose/ketone devices; pharmacist and diabetes specialist-nurse availability; overnight laboratory support
Timeliness indicators	Time to initial assessment; time to first fluid administration; time to insulin initiation; time to potassium reassessment; result-to-clinical-action time
Execution quality	Missed glucose or potassium monitoring rate; delayed-medication rate; pathway deviation rate and reasons; critical-value closure rate; completion of fluid-transition tasks
Transition of care	On-time basal-insulin administration rate; compliance with intravenous-to-subcutaneous overlap; handover completeness; timeliness of first post-transfer monitoring
Safety outcomes	Hypoglycaemia; severe hypokalaemia; fluid-overload events; treatment interruption; unplanned pathway discontinuation; unplanned ICU escalation; mortality
Nursing outcomes	Nursing workload; cognitive burden; pathway acceptability; role clarity; professional autonomy; psychological safety
Patient outcomes	Teach-back completion; self-efficacy; competence in injection and glucose/ketone monitoring; insulin access; follow-up completion; 30-day recurrence
Economic and system outcomes	ICU and ward resource utilisation; point-of-care testing costs; training and pathway-maintenance costs; readmission-related costs

These domains and measures are proposed for future research and implementation evaluation. They have not been validated as a DKA-specific quality-indicator set.

## 5 Information exchange, care transitions, and recurrence prevention

### 5.1 Structured communication, digital collaboration, and team training

Structured communication is most relevant at points where incomplete information can alter treatment, such as critical-value reporting, pathway discontinuation, clinical deterioration, and transfer of care. SBAR may help organise these exchanges, but its usefulness depends on whether the message clearly identifies the patient’s condition, the problem requiring attention, and the action expected from the receiving professional. In DKA care, the Situation component may identify an immediate problem such as worsening acidosis, hypokalaemia, altered consciousness, or inadequate treatment response. Background should summarise relevant diabetes history, precipitating factors, and recent insulin and fluid therapy. Assessment should emphasise trends in glucose, potassium, ketones, acid-base status, and haemodynamic condition, while Recommendation should specify the required action, such as reassessment, treatment adjustment, repeat testing, or escalation of care. However, SBAR is a communication framework rather than a DKA-specific decision tool. Because DKA management depends heavily on serial biochemical and clinical trends, an overly abbreviated SBAR exchange may omit important temporal information. Its effectiveness also depends on staff familiarity, information accuracy, and shared escalation criteria. SBAR should therefore complement, rather than replace, structured DKA handover datasets, critical-value read-back, and closed-loop confirmation of subsequent actions.

Electronic order sets and shared task displays can make current treatment, recent trends, pending tasks, responsibility, and escalation thresholds easier to see. Digital tools can also create new problems, including alert fatigue, duplicate documentation, inconsistent information across systems, and propagation of incorrect data. Their design and testing should involve the staff who use them in routine DKA care, including nurses, physicians, pharmacists, and laboratory personnel. Team training is another part of implementation. Abulebda et al. (35) used *in situ* simulation to assess interprofessional adherence to paediatric DKA guidelines and found considerable variation in institutional capacity and team performance. Waddell et al. (36) introduced interprofessional simulation and feedback based on actual cases in 15 community emergency departments, with improved adherence to best practices. Kempegowda et al. (37) used monthly audit feedback involving emergency, acute medicine, and diabetes teams and reported sustained improvement in several care processes. These studies support repeated simulation, case review, and feedback rather than reliance on a single educational session.AI-assisted clinical decision support is also emerging in emergency and inpatient diabetes care. AI-based systems may help identify patients at risk of dysglycaemia, recognise abnormal glucose patterns, support insulin-related decisions, and prioritise patients or tasks that require clinical review (38). However, evidence for AI-based decision support in emergency care remains largely developmental. In a recent scoping review, Kareemi et al. (39) found that most AI-based clinical decision-support studies in emergency departments remained at an early preclinical stage, with relatively few progressing to advanced testing or routine implementation. In inpatient glycaemic management, Pichardo-Lowden et al. (40) evaluated an electronic alert-based decision-support tool that identified dysglycaemia or inappropriate insulin use and provided management recommendations; use of the active tool was associated with shorter length of stay in several patient groups. These findings suggest possible benefits from earlier risk recognition and more timely treatment adjustment, but they do not establish effectiveness in DKA-specific care. Integration into DKA pathways would require prospective validation, transparent clinical oversight, compatibility with existing electronic systems, and clear responsibility for acting on recommendations. Alert fatigue, inaccurate or incomplete input data, poor generalisability across patient populations, automation bias, and over-reliance on algorithmic recommendations remain important safety concerns. AI-supported recommendations should therefore complement, rather than replace, bedside assessment and interprofessional clinical judgement.

### 5.2 Continuity from the emergency department to the ward and quality improvement

Transfer between the emergency department, observation unit, ward, and ICU is a vulnerable period for treatment interruption. A proposed minimum DKA handover dataset may include disease severity and precipitating factors, recent glucose, potassium, ketone or acid-base results, cumulative fluid input and urine output, current treatment, the timing of basal-insulin administration, the next scheduled monitoring task, outstanding investigations, and findings that may require escalation or pathway discontinuation. Before responsibility is transferred, the sending and receiving teams should confirm outstanding tasks, treatment-transition requirements, and responsibility for subsequent monitoring. During transition from intravenous to subcutaneous insulin, actual administration of basal insulin should be confirmed rather than inferred from an electronic order alone. The receiving unit should also have the capacity to provide the required level of monitoring. Handover completeness, basal-insulin bridging, and the timing of the first post-transfer assessment may serve as candidate measures for evaluating care transitions in future DKA studies, although their validity as DKA-specific quality measures remains to be established. Sharma et al. (41) reported that uptake and consistent implementation of DKA guidelines remained suboptimal in routine practice and that improvements in patient outcomes were not sustained. The DEKODE project further showed that prolonged hospital stay and delayed discharge after DKA were multifactorial, and that changes in documentation or discharge guidance alone did not significantly reduce length of stay (42). For nursing practice, these findings indicate that improving DKA transitions requires more than standardised documentation. Nurses should identify unresolved treatment tasks, confirm readiness for transfer or discharge, verify completion of insulin-transition requirements, and ensure that responsibility for subsequent monitoring is clearly transferred. Nursing assessment should also identify practical barriers to discharge, including incomplete education, uncertainty about insulin use, lack of supplies, and unresolved follow-up arrangements. When avoidable workflow barriers contribute to delay, nurses may participate in escalation and multidisciplinary review. Overall, written guidance is unlikely to change practice without coordinated attention to education, workflow design, audit, and feedback.

### 5.3 Multidisciplinary discharge preparation and recurrence prevention

Resolution of DKA does not mean that the patient’s subsequent risk has resolved. In a systematic review and meta-analysis of DKA readmission, Ji et al. (43) grouped associated factors into demographic, socioeconomic, diabetes-related, comorbidity, and health-behaviour domains; low income, mental health disorders, and discharge against medical advice were among the more consistent factors. In a scoping review, Mohler et al. (44) found that factors associated with DKA readmission spanned clinical status, health behaviours, and the social environment, suggesting that readmission risk may arise from multiple interacting influences. Management of high-risk patients should therefore extend beyond disease control to include interprofessional risk screening, individualised intervention, and continuing post-discharge follow-up. Shaka et al. (45) analysed US admissions for DKA in people with type 1 diabetes and identified several comorbid and social-behavioural factors associated with 30-day readmission. Lyerla et al. (46) further reported that housing instability, substance use, mental health problems, and barriers to healthcare access contributed to recurrent DKA. Hamblin et al. (47) argued that DKA may be an important signal of mental health disorders and difficulties with diabetes management. These findings indicate that emergency and specialist nurses should begin recurrence-risk screening once the patient’s condition is stable rather than postponing psychosocial assessment until the day of discharge. Discharge preparation may require physicians, pharmacists, bedside nurses, diabetes specialist nurses, and, when indicated, social workers and mental health professionals. Physicians explain precipitating factors and long-term treatment goals; pharmacists review prescriptions, storage, and medication access; specialist nurses assess injection and glucose/ketone monitoring skills; bedside nurses use teach-back to confirm understanding of sick-day management and indications for seeking care; and social support professionals address financial, housing, family-support, and follow-up barriers. Patients should be active participants in discharge planning rather than passive recipients of instructions. Shared decision-making should incorporate the patient’s treatment preferences, self-management capacity, psychosocial circumstances, and anticipated barriers after discharge (48). With the patient’s consent and where appropriate, family members or other caregivers should also be involved in education and planning, particularly when they assist with insulin administration, glucose or ketone monitoring, recognition of warning signs, or access to emergency care (49). Family involvement should support, rather than replace, patient autonomy and individualised decision-making. Simmons et al. (50) evaluated a combined diabetes specialist and mental health team intervention and suggested that comprehensive assessment and support may reduce readmission among patients with recurrent acute glycaemic events. A scoping review by Cai et al. (51) concluded that multidisciplinary and dedicated care teams may help reduce preventable diabetes-related readmissions.

The quality of discharge should not be judged solely by documentation that education was provided. Evaluation may include whether the patient can independently perform injection and monitoring, explain the action plan using teach-back, obtain insulin and supplies before discharge, attend a scheduled follow-up appointment, and be contacted successfully soon after discharge. Stratified follow-up should be considered for recurrent DKA, newly diagnosed diabetes, adolescents in transition, difficulty obtaining medication, and high psychosocial risk. Information on recurrence should be fed back to emergency and diabetes teams to inform the next cycle of quality improvement.

For patients using SGLT2 inhibitors or with a history of euDKA, sick-day education should specifically address when to withhold medication, when to monitor ketones despite near-normal glucose levels, how to maintain hydration and carbohydrate intake when appropriate, and when to seek urgent medical assessment.

## 6 Limitations of the current evidence and key controversies

### 6.1 Limited direct evidence for complete interprofessional nursing models

Current studies of emergency DKA nursing mainly evaluate nurse-led tools, standardised pathways, non-ICU management, telepharmacy services, diabetes specialist-nurse services, and interinstitutional training. Few studies have treated a complete team comprising emergency nurses, emergency physicians, endocrinologists, pharmacists, laboratory personnel, and specialist nurses as an independent intervention. Reductions in hypoglycaemia, shorter emergency department stays, or lower ICU use after pathway implementation may be influenced simultaneously by patient selection, electronic order sets, staff education, unit structure, and bed-management policies, making the independent contribution of collaboration difficult to determine.

General evidence on interprofessional collaboration, emergency nurse-initiated pathways, and diabetes care transitions can help explain implementation mechanisms but remains indirect and cannot replace DKA-specific research. Few studies have directly measured team role clarity, shared decision-making, information exchange, critical-value closure, or the psychological safety required for nurses to challenge or pause a pathway. Current evidence supports the feasibility of standardised pathways and individual professional services but does not establish the optimal composition, hours of availability, or division of responsibilities within a complete team. Importantly, the closed-loop framework proposed in this review represents a conceptual synthesis rather than an empirically validated intervention. Although several individual components are supported by DKA-specific studies, the relationships among the six stages, the proposed role configuration, and several nursing-sensitive process indicators remain hypothesis-generating and require prospective evaluation. This review also has limitations inherent to a narrative review design. Although a structured multi-database search and predefined eligibility approach were used, the review was not conducted as a registered systematic review and did not include formal dual-reviewer screening, systematic risk-of-bias assessment, or quantitative evidence synthesis. Relevant studies may therefore have been missed, and study selection and interpretation remain susceptible to selection bias and reviewer judgement. Publication bias may also favour reports of successful pathway implementation. The proposed framework should therefore be interpreted as an evidence-informed conceptual synthesis rather than an exhaustive or quantitatively weighted representation of the literature.

### 6.2 Nursing workload and resource controversies in non-ICU pathways

Reduced ICU utilisation does not necessarily imply lower nursing resource requirements.

Non-ICU pathways may shift time-sensitive tasks, including medication administration, repeated point-of-care testing, blood sampling, and scheduled reassessment, to general wards where nurse-to-patient ratios are often higher. If staffing and workflow are not adjusted accordingly, missed monitoring, delayed treatment, or delayed recognition of deterioration may offset the operational benefits of avoiding ICU admission. Economic evaluations may likewise underestimate the true cost of non-ICU care when nursing time, staff training, point-of-care testing, electronic-system maintenance, and interunit coordination are not included. Safe implementation therefore requires explicit assessment of nursing workload, workflow capacity, and unit readiness before pathway introduction. Practical strategies include defining patient eligibility criteria, mapping time-sensitive nursing tasks, establishing minimum monitoring requirements, specifying escalation procedures, and adjusting staffing when protocol-related task intensity exceeds usual ward capacity. Pilot implementation accompanied by audit of missed monitoring, delayed medication, unplanned escalation, and nursing workload may help identify unsafe workflow before wider adoption. Electronic task prompts and point-of-care testing may improve timeliness, but their effects on documentation burden and cognitive workload should also be evaluated. Future studies should use prospective multicentre designs where feasible and incorporate balancing measures alongside clinical outcomes. Cluster-randomised, stepped-wedge, or mixed-methods implementation designs may be particularly useful for evaluating both effectiveness and workflow consequences. Nursing workload should be measured directly using nurse-to-patient ratios, task-frequency data, time-motion methods, missed-care measures, or validated workload instruments. Staff acceptability, workflow disruption, implementation fidelity, and reasons for pathway deviation should also be assessed to determine whether lower ICU use reflects genuine efficiency or redistribution of work to less intensively staffed settings. Organisational differences also limit the generalisability of current non-ICU pathway studies. A pathway that is feasible in a centre with adequate nurse staffing, rapid laboratory turnaround, specialist support, and immediate access to higher-acuity care may not be equally safe in settings with fewer staff, slower laboratory services, or limited escalation capacity. Local implementation should therefore be preceded by assessment of both patient eligibility and institutional readiness.

### 6.3 Study design, outcome measures, and external validity

Most DKA pathway studies are single-centre retrospective cohorts, before-after comparisons, or historical-control studies and are vulnerable to confounding by case severity, bed policy, staffing, and concurrent quality-improvement initiatives. Even large database studies often lack nursing-process and team-behaviour data, limiting understanding of the mechanisms underlying outcome changes. Inconsistent definitions of DKA resolution and pathway eligibility further impede comparison across studies. External validity is also limited for special populations. Studies of non-ICU pathways for mild-to-moderate DKA commonly exclude pregnant patients, children, patients with severe cardiac or renal impairment, altered consciousness, or euglycaemic DKA. Transferability may also depend on specific healthcare-system characteristics, including nurse-to-patient ratios, legal and organisational scope for nurse-initiated care, availability of observation units and ICU beds, access to point-of-care testing and rapid laboratory services, 24-hour pharmacy or diabetes-specialist support, electronic health-record interoperability, and the speed of medical or critical-care escalation. Discharge practices may also vary according to medication and supply coverage, access to diabetes education, outpatient follow-up capacity, and availability of mental-health and social-support services. Differences in these characteristics may substantially alter both the feasibility and safety of the same DKA pathway across hospitals and healthcare systems. Limited measurement of nursing-sensitive and patient-reported outcomes means that current research cannot adequately determine whether pathways improve patient experience, increase nurses’ cognitive workload, or prepare patients for self-management after discharge.

General implementation studies of nurse-initiated protocols indicate that workload, competence, documentation, resources, and nurse-physician relationships jointly influence implementation (20, 21). These findings can inform DKA pathway strategies but require validation in DKA-specific settings. Pathway failure should not be attributed simply to individual non-adherence; knowledge or skill deficits, poorly designed tasks, inadequate organisational resources, information-system problems, and clinically appropriate deviations for special populations should be distinguished. The barriers, optimisation strategies, and outcomes relevant to emergency interprofessional DKA nursing are summarised in **Figure 2**.

**Figure 2:**
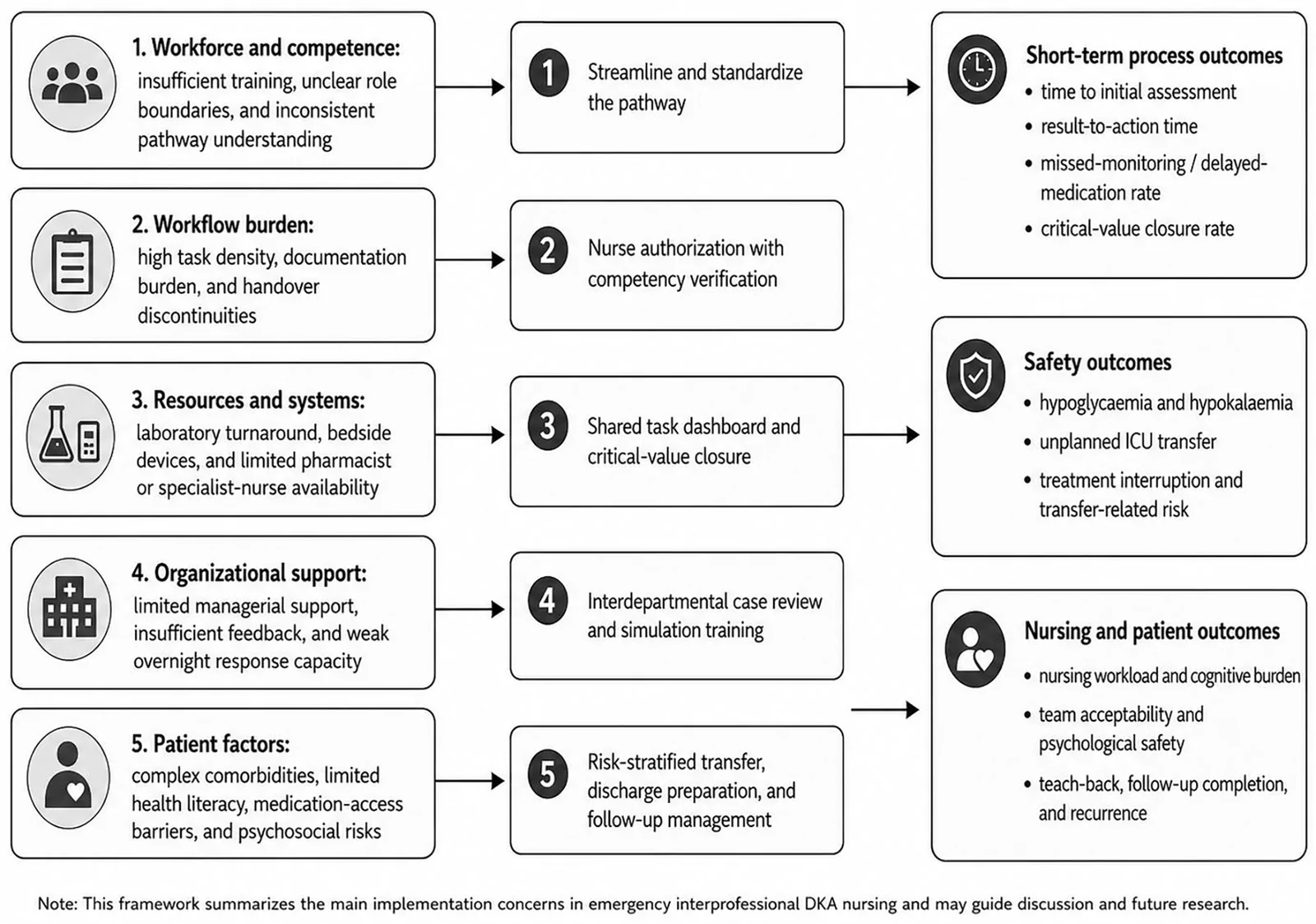
Implementation barriers, optimization strategies, and outcome framework for emergency interprofessional DKA nursing.

## 7 Future research directions and conclusions

### 7.1 Future research directions

Future studies should evaluate the complete interprofessional DKA pathway rather than continuing to examine isolated treatment components alone. Multicentre cluster-randomised trials, stepped-wedge designs, interrupted time-series studies, and controlled quality-improvement programmes could be used to examine team composition, the limits of nurse authorisation, pharmacist and specialist-nurse availability, laboratory standards, receiving-unit requirements, escalation procedures, patient selection, and implementation fidelity. The proposed closed-loop framework provides a structure for this research agenda. For recognition and activation, future studies should evaluate the timeliness and reliability of early DKA identification and pathway activation. For shared risk stratification, research should examine agreement on care-setting decisions and the safety of risk-based patient selection. For coordinated monitoring and execution, studies should assess result-to-action time, completion of scheduled monitoring, medication delays, and critical-value closure. For escalation, research should evaluate trigger recognition, response time, and unplanned transfer to higher levels of care. For care transition, studies should assess handover completeness, treatment continuity, basal-insulin bridging, and post-transfer monitoring. For recurrence prevention, research should examine patient and family participation, self-management readiness, follow-up completion, and recurrent DKA. These stage-specific measures should also be linked to overall safety, nursing workload, patient experience, and resource use. A DKA-specific core outcome set for interprofessional emergency nursing has not yet been established. The domains proposed in this review provide candidate areas for future evaluation, including clinical safety, timeliness of care, task execution, care transition, nursing workload, team functioning, patient self-management, recurrence, and resource use. Development of such a core outcome set should explicitly map candidate measures to the six stages of the proposed framework and distinguish stage-specific process measures from patient-level and system-level outcomes. Before these measures are adopted for benchmarking or formal quality assessment, their feasibility, reliability, validity, responsiveness, and associations with clinical outcomes should be examined across different settings. Mixed-methods research may be particularly useful because quantitative outcomes alone cannot explain why the same pathway works in one setting but not another. Interviews and field observation can examine workflow, professional relationships, use of information systems, and patient experience alongside clinical and operational outcomes. Gawthorne et al. (52) used behaviour-change theory to develop an implementation strategy for emergency nurse-initiated protocols and identified education, clinical champions, documentation optimisation, audit and feedback, and leadership support as relevant components. These factors are also relevant when adapting DKA pathways to local practice. Resource availability will affect implementation. Centres with greater resources may have point-of-care ketone testing, digital task systems, and continuous pharmacist or specialist-nurse support, whereas other hospitals may rely more on simplified protocols or remote support. Patient-selection criteria, escalation arrangements, and safe treatment transition should remain explicit regardless of the setting. Future economic evaluations should consider nursing time, training, point-of-care testing, pharmacy support, and system-maintenance costs in addition to ICU and ward resource use.

## 8 Conclusions

Emergency DKA nursing is a time-dependent, information-intensive, and highly interdependent process. Quality depends not only on whether the clinical plan is guideline concordant but also on whether early risk is recognised, key assessments are completed on time, abnormal results trigger accountable action, and care remains continuous across the emergency department, inpatient ward, and post-discharge period. Emergency nurses have a central coordinating role in continuous assessment, bedside information integration, pathway implementation, and risk escalation; however, nurse-led care must be supported by explicit authorisation, targeted training, appropriate staffing, and timely medical input.

Current evidence suggests that standardised emergency pathways, nurse decision-support tools, interdepartmental quality-improvement initiatives, pharmacist involvement, laboratory support, diabetes specialist-nurse services, and interprofessional simulation and feedback can improve selected process, safety, and resource outcomes. Nevertheless, direct evaluations of complete emergency interprofessional DKA nursing teams remain limited, and many conclusions are constrained by single-centre designs, historical controls, and co-interventions. Future research should move beyond comparisons of isolated treatment components to evaluation of complete collaborative systems and should simultaneously address task timeliness, closed-loop communication, workload, team psychological safety, patient experience, care transitions, and long-term recurrence.

The defining feature of interprofessional nursing is not the number of professions involved but the maintenance of the accountable chain defined in the proposed closed-loop framework, in which information is recognised, responsibility is assigned, actions are executed and confirmed, effects are reassessed, and escalation occurs as the patient’s condition changes. Only by translating interprofessional collaboration into clear, actionable, measurable, and continuously improvable emergency nursing processes can the safety, continuity, and resource efficiency of DKA care be enhanced.

## Data Availability

The original contributions presented in the study are included in the article/supplementary material. Further inquiries can be directed to the corresponding author.
